# Patella Osteomyelitis Mimicking Sinding-Larsen and Johansson Apophysitis: A Pitfall Not to Miss

**DOI:** 10.1155/2020/1780689

**Published:** 2020-12-07

**Authors:** Aurélien Traverso, Benjamin Tschopp, Tristan Mekdade, Barbara Kwiatkowski, Nicolas Lutz

**Affiliations:** Department of Pediatric and Adolescent Surgery, Lausanne University Hospital (CHUV) and University of Lausanne (UNIL), Lausanne, Switzerland

## Abstract

**Background:**

Diagnosis of bony infection remains difficult during childhood. Osteomyelitis of the patella (OMP) is rare and produces few symptoms and no fever. A high level of suspicion is needed to avoid missing this uncommon type of bone infection.

**Methods:**

/

**Results:**

We report an acute osteomyelitis of the patella treated by joint and patella puncture aspiration followed by antibiotics in a 12-year-old healthy boy. Clinical and radiological findings, orthopedic follow-up, and clinical outcome are presented.

**Conclusion:**

Making a diagnosis of acute osteomyelitis of the patella can be challenging especially in the pediatric population, as it produces few specific symptoms with mostly nonspecific biologic anomalies. The paucity in specific signs and symptoms, accompanied by the rare occurrence, often leads to incorrect initial diagnosis, such as overuse injury or apophysitis. Clinical examination with high suspicion for patella infection is key. Radiological exams including MRI are the main components of the adequate imaging studies. Computed tomography may be an excellent addendum to better visualize any bony lesion within the patella. Bone aspiration or biopsy is essential to confirm the diagnosis and offers a first step in the treatment of this infection, which can then be successfully treated with a normal course of antibiotics.

## 1. Introduction

Osteomyelitis (OM) during growth has a prevalence of 2.9 per 100,000 children, with declining values in “developed” nations [1]. The first case of acute osteomyelitis of the patella (OMP) was described by Thirion in 1829 [2]. Reaching the diagnosis can be challenging, due to the paucity of its nonstandard clinical presentation [3]. Involvement of the patella in OM is very uncommon, when compared to the typical long bones localization [4, 5]. To establish with certitude, the diagnosis of a musculoskeletal infection remains an invasive process. Standard radiographs may be normal or reveal nonspecific cortical condensation, erosion, or periosteal reaction. When present, they are usually secondary to a significant delay between the beginning of the infection and the diagnosis. Magnetic resonance imaging (MRI) is the gold-standard imaging study for the diagnosis of osteomyelitis [6]. MRI can demonstrate bone edema or periosteal inflation, abscesses, and possible adjacent joint effusion. It can delineate any extraosseous spread of the disease. Aspiration is mandatory for the identification of the causative agent.

Early diagnosis of OM in children is the key to implement adequate antibiotic treatment and prevent severe sequelae such as bone or joint destruction, which can produce lifelong disabilities [3].

The authors report on a case of acute osteomyelitis of the patella (OMP) in a healthy 12-year-old boy treated successfully by local aspiration followed by a course of intravenous and oral antibiotics. Clinical presentation and radiological findings mimicking the Sinding-Larsen and Johansson apophysitis are described. The investigation process and treatment modalities are explained and discussed, comparing them to published case reports.

## 2. Statement of Informed Consent

The patient and his parents were informed that data concerning the case would be submitted for publication. The patient and parents agreed.

## 3. Case Report

**Figure 1:**
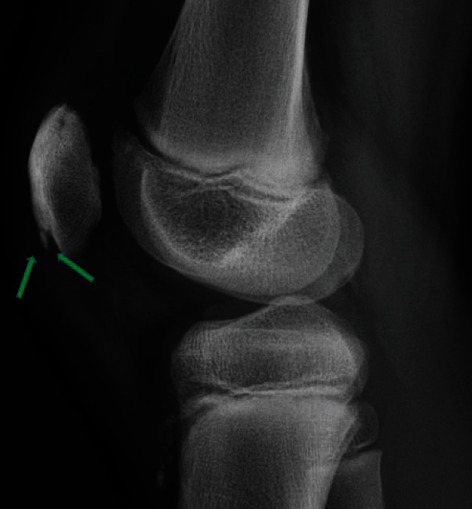
X-ray 1. Lateral view of the right knee. Green arrows shows the Sinding-Larsen and Johansson disease, initial radiological findings.

**Figure 2:**
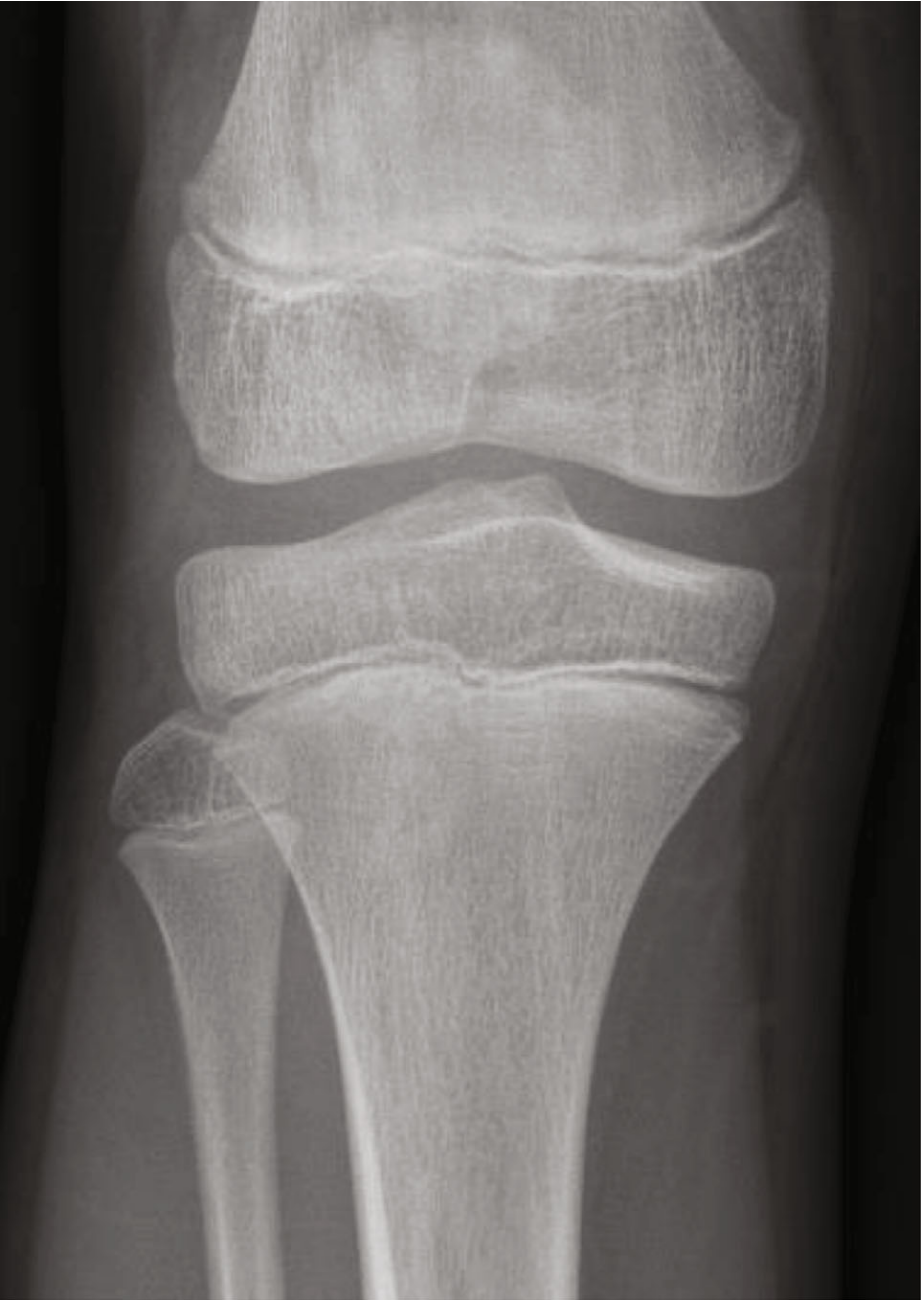
X-ray 2. AP view of the right knee, initial radiological findings.

**Figure 3:**
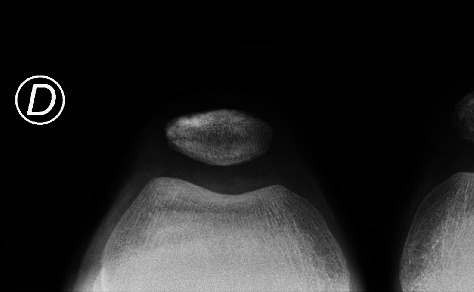
X-ray 3. Axial view of both knees (right knee on the left), initial radiological findings.

A 12-year-old boy presented with a painful and swollen knee. He described anterior knee pain when walking and standing which had begun 48 hours prior to consultation. There was no history of acute fever, past trauma, or rheumatological history. Physical examination revealed antalgic gait and pain during compression of the inferior pole of the patella, mimicking a Sinding-Larsen and Johansson disease (SLJD), with limitation of the range of motion: active 140°-20°-0° (passive 140°-0°-0°). Full flexion of the knee was painful. The extensor apparatus was competent. Initial diagnosis was a SLJD due to localization of the symptoms and initial radiological findings on X-rays. Figures 1–3 (green arrows) show SLJD typical X-rays with calcifications plus irregular ossification of the lower pole of the patella and thickening and heterogeneity of the proximal patellar tendon. Blood tests showed a C-reactive protein (CRP) of 64 mg/l, sedimentation rate (SR) of 15 mm/h, and a normal leucocytes count (WBC) 72 hours after the beginning of the symptoms. The patient was treated with nonsteroidal antiinflammatory drugs (NSAIDs) and a brace for immobilization.

24 hours after initial diagnosis, the patient was reassessed as scheduled. There were no changes in the physical exam. CRP increased to 96 mg/l. The patient remained fever free.

**Figure 4:**
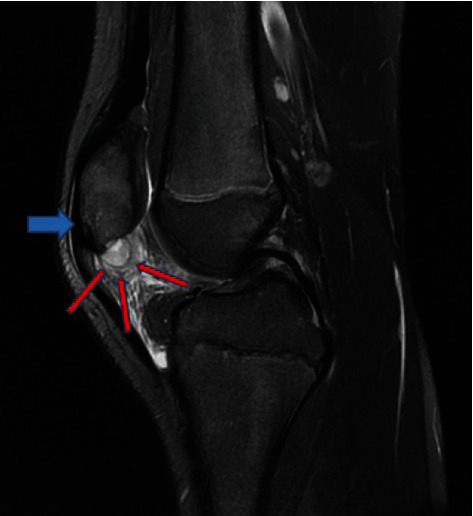
MRI 1. Sagittal T2 fat sat view. Osteomyelitis of the patella (blue arrow) and fat pad infiltration were observed (red arrows).

**Figure 5:**
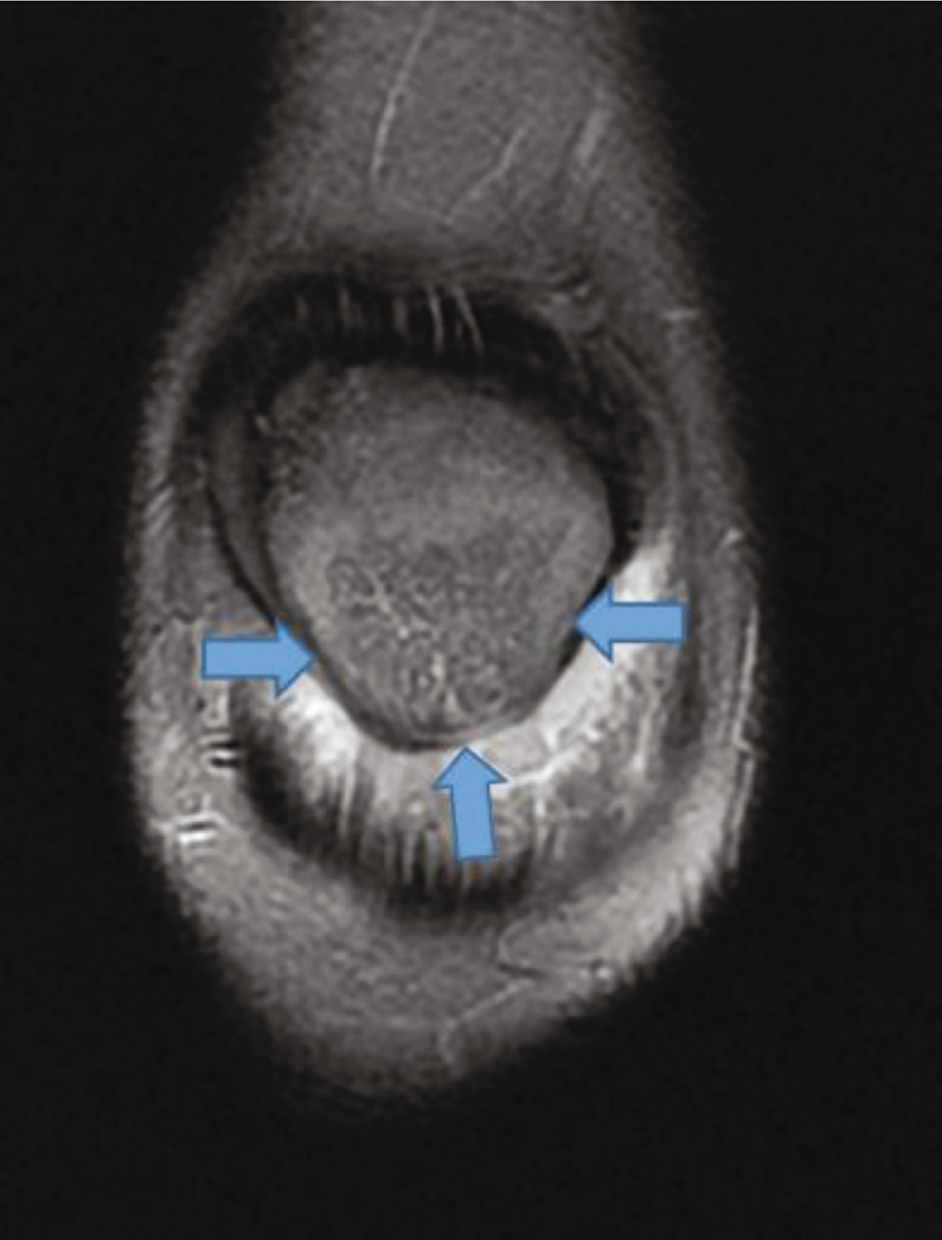
MRI 2. Coronal T2 fat sat view. Osteomyelitis of the patella (blue arrows).

**Figure 6:**
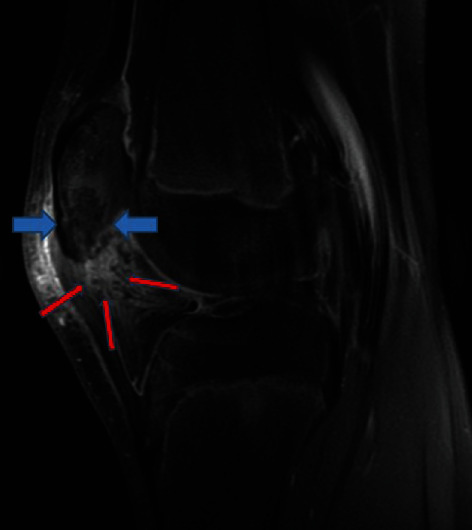
MRI 3. Sagittal T1 fat sat view. Osteomyelitis of the patella (blue arrows) and fat pad infiltration were observed (red arrows).

Because of the increased CRP, an infectious process and a magnetic resonance imaging (MRI) were performed, and the suspected diagnosis of OMP was established (Figures 4–6, blue arrows). An abscess of Hoffa's fat pad with circular fat pad infiltration was observed (Figures 4–6, red arrows). A puncture aspiration of the knee and the patella was performed in the operating theatre, under general anaesthesia, using a 17-gauge needle. First, a joint aspiration from a superolateral approach was performed, yielding a synovial fluid of physiological appearance, Then, a punction of Hoffa's fat pad and intraosseous lesion at the lower pole of the patella were performed, which yielded 1.5 ml of sanguine-purulent liquid. Directly after having collected the samples, empiric antibiotics were started intravenously (amoxicillin/clavulanate 50 mg/kg 3 times per day). The culture of the patella punction revealed a *Staphylococcus aureus* and so confirmed the presupposed diagnosis (>10 0000 germs/ml, methicillin sensible). Articular fluid analysis showed an inflammatory state with predominantly neutrophil polymorphonuclear cells. Two days after the puncture aspiration and the beginning of antibiotics, biological tests showed a significant decrease of the CRP at 58 mg/l and normal WBC count. Antibiotics were given intravenously for 5 days then changed for oral antibiotics (amoxicillin/clavulanate 30 mg/kg 3 times per day). At discharge, the patient was pain free with simple painkillers. The knee was immobilized in a full-extension brace for 15 days. Antibiotics were given for a total of four weeks according to Wagner et al. [7].

One week after the diagnosis, the follow-up examination was satisfactory, and the patient was pain free.

**Figure 7:**
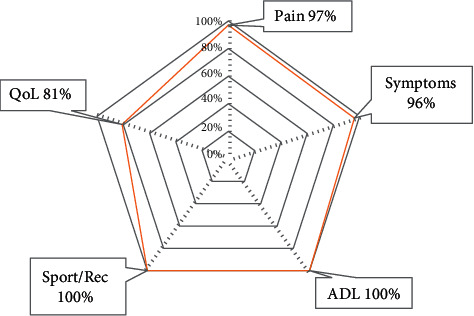
KOOS-child knee survey at 6 weeks postoperative.

**Figure 8:**
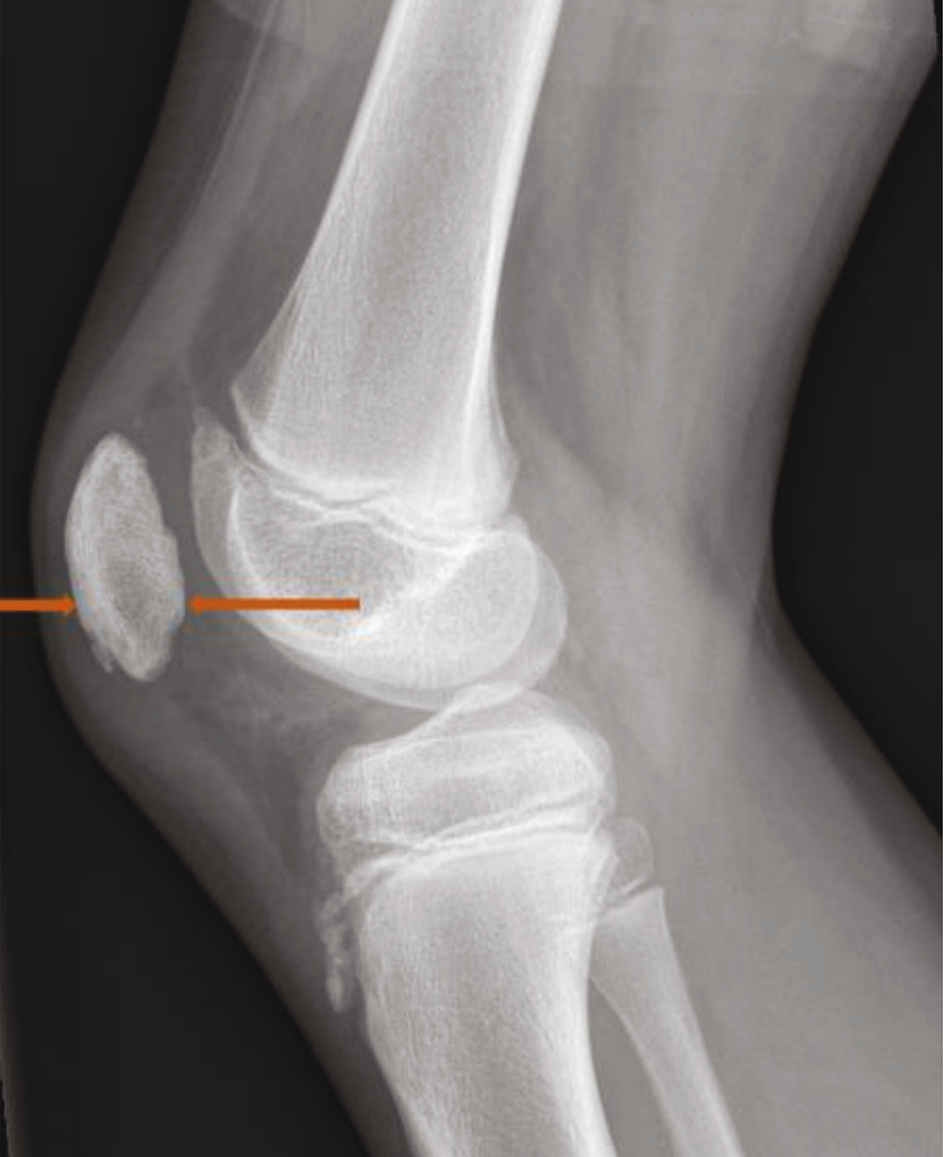
X-ray 4. Lateral view of the right knee, 6 weeks after diagnosis. Orange arrows show the lysis area at the lower pole of the patella.

**Figure 9:**
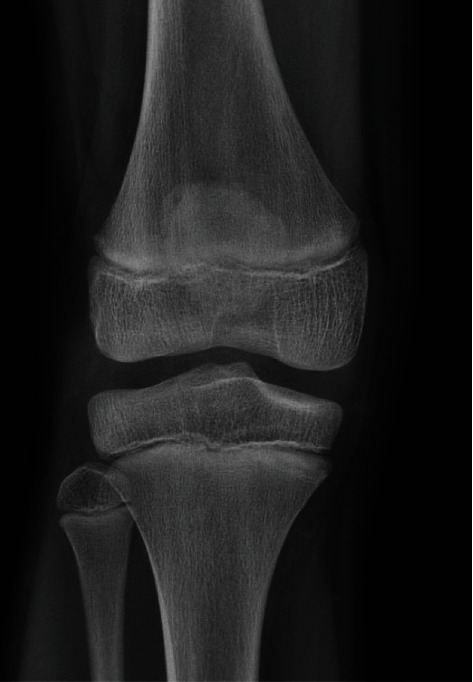
X-ray 5. AP view of the right knee, 6 weeks after diagnosis.

The six-week follow-up examination showed a full and symmetric range of motion of the knee (140°-0°-0°) without any pain neither during active mobilization. The patient reported return to daily life activities and was able to hop on the affected leg and run without pain. Knee Osteoarthritis Outcome Score (KOOS) (see Figure 7) and the Lower Extremity Functional Scale (LEFS) at 79/80 points were excellent. The radiograph of the knee showed a bone lysis area at the lower pole of the patella (Figures 8 and 9, orange arrows show the lysis area).

Clinical assessment at 6 months showed a normal knee function. KOOS and the LEFS score were both at the maximum.

There were no immediate or middle-term complications in this case.

## 4. Discussion

Case series and review of the literature suggest a hematogenous origin for osteomyelitis of the patella, such as in this case. No local skin injury was recorded [8]. Its peak of prevalence seems to be between 5 and 15 years of age [4, 9–14]. Before the age of 5, the patella is primarily cartilaginous, which can explain the extremely low prevalence of OMP in the first years of life [11, 15]. The significant vascularisation, provided by an anastomotic extraosseous arterial network composed with the anterior tibial recurrent artery and the superior and inferior geniculate arteries, begins at the age of 4 to 5 [8]. The patella reaches its maximal blood supply by age 12. Radiological evidence of OMP may appear at a later stage of the disease because the patella is covered by a thin lamina with no real periosteum [12]. Therefore, the typical periosteal elevation found in other bone localizations may be absent in patellar infections [5].

Medical history should be reviewed accurately to detect congenital anomalies or past history of infection. The clinical exam, however, is the key when suspicion arises for OMP. Comparison of both sides is mandatory and can reveal asymmetries.

Septic arthritis of the knee is often suspected initially, which can delay the diagnosis of OMP. Synovitis, septic bursitis, and peripatellar cellulitis can also be confused whit OMP [9]. Differential diagnoses of patellar lesions, such as the Sinding-Larsen and Johansson disease, bipartite patella, and a variety of rare tumors (chondroblastomas, giant cell tumors, osteoid osteomas, and aneurysmal bone cysts) could mislead the surgeon [16].

Routine laboratory tests usually show normal WBC count even in the setting of acute osteomyelitis. SR and CRP are often elevated; however, they both lack specificity in the absence of other radiologic and microbiologic analysis. In cases of proven osteomyelitis, both tests (especially CRP) may be used to assess response to therapy or relapse [17].

Radiographs need to be performed as the first-line imaging. Computed tomography (CT) and magnetic resonance imaging (MRI) can be of great value in the diagnosis and evaluation of osteomyelitis. They show anatomic details, including cortical destruction and soft tissue extension of the infection which can be helpful to choose between a less (punction, biopsy) and a more invasive diagnostic and therapeutic act (open drainage and curettage) [17].

*Staphylococcus aureus*, like in this case, seems to be the most commonly found infecting organism [3, 5, 18].

No consensus exists concerning the optimal OMP treatment. In some simple cases, antibiotics may even be enough to treat OMP [3, 18]. The preferred routes of administration and the duration greatly vary between the different cases described in the litterature [3]. Debridement and curettage as surgical treatment represent the recommended therapy for OMP in case of absence of improvement despite antibiotic treatment [3, 4, 18]. There is clearly no evidence for arthrotomy in cases without joint involvment [3]. The punction aspiration as performed in this case is less invasive and offers a way to decompress eventual bone abscess and to withdraw sample for microbiologic analysis in order to get the exact diagnosis. It is also possible to make an articular punction of the knee in the same time when concomitant arthritis is suspected. It should be performed in the operating theatre under strict aseptic conditions, under general anaesthesia and fluoroscopy.

Diagnose and treatment of OMP provide challenges for the clinician as was shown in this case.

## 5. Conclusion

OMP continues to be a challenging diagnosis in children, especially with regard to unusual bacteria as well as unusual locations. In the emergency department, limping children without fever may still be affected by an uncommon OMP. A high grade of suspicion should arise for any painful knee with a slight effusion and mild ESR and CRP elevation. MRI remains the gold-standard imaging studies in such circumstances.
